# Peroxisomes in Immune Response and Inflammation

**DOI:** 10.3390/ijms20163877

**Published:** 2019-08-08

**Authors:** Francesca Di Cara, Pierre Andreoletti, Doriane Trompier, Anne Vejux, Margret H. Bülow, Julia Sellin, Gérard Lizard, Mustapha Cherkaoui-Malki, Stéphane Savary

**Affiliations:** 1 Department of Microbiology and Immunology, Dalhousie University, IWK Health Centre, Halifax, NS B3K 6R8, Canada; 2 Lab. Bio-PeroxIL EA7270, University of Bourgogne Franche-Comté, 6 Bd Gabriel, 21000 Dijon, France; 3 Molecular Developmental Biology, Life & Medical Sciences Institute (LIMES), University of Bonn, 53115 Bonn, Germany

**Keywords:** peroxisome, immune response, immunometabolism, virology, phagocytosis, inflammation

## Abstract

The immune response is essential to protect organisms from infection and an altered self. An organism’s overall metabolic status is now recognized as an important and long-overlooked mediator of immunity and has spurred new explorations of immune-related metabolic abnormalities. Peroxisomes are essential metabolic organelles with a central role in the synthesis and turnover of complex lipids and reactive species. Peroxisomes have recently been identified as pivotal regulators of immune functions and inflammation in the development and during infection, defining a new branch of immunometabolism. This review summarizes the current evidence that has helped to identify peroxisomes as central regulators of immunity and highlights the peroxisomal proteins and metabolites that have acquired relevance in human pathologies for their link to the development of inflammation, neuropathies, aging and cancer. This review then describes how peroxisomes govern immune signaling strategies such as phagocytosis and cytokine production and their relevance in fighting bacterial and viral infections. The mechanisms by which peroxisomes either control the activation of the immune response or trigger cellular metabolic changes that activate and resolve immune responses are also described.

## 1. Introduction

Peroxisomes are almost ubiquitous metabolic organelles conserved across the eukaryotes. Peroxisomes were identified as the cellular compartment where the turnover of some reactive anionic species and complex lipids take place. The metabolic functions of peroxisomes are closely associated with mitochondrial metabolism [1,2,3]. For example, peroxisomal very long-chain fatty acids (VLCFAs) have to be degraded by the peroxisomal β-oxidation enzymes but the resulting shortened acyl-CoAs serve as substrate for the mitochondrial carnitine shuttle to be fully oxidized into the mitochondria. In yeast, the physical interaction between the two organelles has recently been demonstrated and shown to be important for fatty acid β-oxidation [4]. Moreover, both organelles tightly cooperate to control the redox homeostasis in the cell [1,5]. In addition, mitochondria and peroxisomes share the proteins Fis1, Mff and DLP1/Drp1 for fission [6]. To date, multiple studies have focused on dissecting the role of mitochondria as signaling organelles participating in different cellular processes. For instance, many studies have demonstrated that mitochondria are major hubs of the immune and inflammatory response, regulate cell death, and are important in signaling and intracellular compartmentalization processes which protect against a variety of pathogens [7,8]. Peroxisomes, which share many features with mitochondria, have paradoxically long been overlooked.

For many years, peroxisomes have been considered metabolic organelles with no other vital cell functions [9]. The idea that peroxisomes are involved in regulating inflammation and antimicrobial responses emerged only a few years ago and originated from three important properties attributed to these organelles: (i) They can produce and clean up cellular reactive anionic species such as reactive oxygen species (ROS) and reactive nitrogen species (RNS) [10], thereby contributing to the cellular redox status [11]; (ii) they are involved in the degradation of prostaglandins [12] and leukotrienes [13,14], which are primary modulators of inflammation [15]; and (iii) they are involved in polyunsaturated fatty acid metabolism, being associated both with their degradation and with their synthesis [16]. Peroxisomal metabolism is indeed the main endogenous supplier of docosahexaenoic acid (DHA) and contributes to the control of eicosapentaenoic acid (EPA) and docosapentaenoic acid (DPA) levels [17,18]. These polyunsaturated fatty acids (PUFAs) are the backbone of a series of mediators for the resolution of inflammation such as resolvins, maresins, and protectins [19]. Notably, reactive anionic species play a central regulatory role in various aspects of the immune response, such as host defense, interaction and activation of innate and adaptive immune cells, and immune suppression [20,21,22,23,24,25]. Similarly, lipids such as fatty acids regulate inflammatory processes [25,26,27,28] and are signaling molecules for the activation and function of macrophages, invariant Natural Killer T (iNKT) cells and T-cells [29]. Therefore, it is not surprising that considerable experimental evidence has recently emerged to support the idea that peroxisomes are pivotal organelles in orchestrating cellular and systemic immune response strategies by serving as signaling platforms to initiate immune pathways, and by controlling the synthesis and breakdown of immune bioactive metabolites [30,31,32,33].

This review first summarizes the peroxisomal proteins and peroxisomal metabolites that have been suggested or demonstrated to function in different immune cells, thus supporting a role of peroxisomes in pro- and anti-inflammatory signaling governing the immune response. Secondly, following examples in human peroxisomal disorders and corresponding animal models, evidence is reported for a role of peroxisomes in inflammation control during neurodegeneration and aging. Then, recent data is presented supporting the role of peroxisomes in antiviral immunity, making these organelles possible therapeutic targets to control infection. Finally, this review focuses on the recent discovery that peroxisomes play a major role in controlling phagocytosis, an important innate immunity process involved in the modulation of inflammation and tissue remodeling.

## 2. The Regulation of Peroxisomal Genes is Correlated to Proliferation and Activation of Various Immune Cells

Recent evidence has demonstrated that the role of the immune system extends far beyond the interaction with pathogens, and that it acts also as a sensory system to maintain tissue development and homeostasis. Thus, an imbalanced immune response results in disorders of immunodeficiency or autoimmunity, or in chronic inflammation that affects development and tissue homeostasis. Immune disorders and chronic inflammation encompass a wide spectrum of human diseases with an ever-increasing impact on health. There is therefore a pressing need to characterize the underlying networks that govern immune cell function in both health and disease. The success of an immune response involves distinct signaling pathways in various CD34+ derived cell types of myeloid origin (dendritic cells, macrophages, neutrophils, eosinophils, basophils) or lymphoid origin (natural killer cells (NK), natural killer T cells (NKT), T helper and T cytotoxic lymphocytes, B lymphocytes and plasmacytoid dendritic cells) and in various tissues (bone marrow, thymus, lymph nodes, spleen, tonsils and other specialized tissues (mucosa-associated lymphoid tissues, MALT)). Many of these signaling pathways are tightly connected to cellular metabolism, and mitochondrial signals have been found essential in immune cells [8]. Peroxisomes display very specific metabolic roles but also overlapping functions with mitochondria. These two organelles interact, and it is therefore not surprising that peroxisomes also play an important role in the function of immune cells.

Table 1 summarizes the proteins for which experimental evidence has proven peroxisomal localization in the immune tissues or cells and has suggested their possible involvement in immune response and inflammation. Their involvement concerns not only the control of immune cell proliferation, but also immune cell activation or their involvement in antiviral responses. All of the eighteen identified proteins are involved in well-known peroxisomal pathways (lipid metabolism, VLCFA β-oxidation, antioxidant system, ether lipids synthesis, α-oxidation and peroxisome biogenesis).

Some of these proteins have been suggested to control or interfere with immune cell proliferation. For instance, an analysis from the Oncomine database to determine gene alterations during carcinogenesis, revealed a strong cluster of 144 genes across a panel of 1995 leukemia and 74 normal blood tissues. Among this cluster, *ACSCL6* was identified as a potential tumor suppressor gene in leukemia [34]. The *ACSCL6* gene encodes for a peroxisomal acyl-CoA synthetase converting VLCFAs into acyl-CoA thioester, a required step for their transport into the peroxisomal matrix by the peroxisomal ABC transporters. Indeed, a unique case of chronic eosinophilic leukemia with a novel t(5;12) (q23–31;p13)/*ETV6-ACSL6* gene fusion where the patient was resistant to tyrosine kinase inhibitor therapy was recently reported [35]. Another example concerns the partially peroxisome-localized enzyme isocitrate dehydrogenase 1 (IDH1), which catalyzes the oxidative decarboxylation of isocitrate to generate α-ketoglutarate and CO_2_ (glyoxylate metabolism) [36]. It is suggested that this activity is linked to the peroxisomal regeneration of nicotinamide adenine dinucleotide phosphate (NADPH) using a specific 2-ketoglutarate/isocitrate transporter of the peroxisomal membrane to transfer redox equivalents [37]. In addition, the peroxisomal form of IDH1 was shown to provide 2-oxoglutarate as substrate to the peroxisomal phytanoyl-CoA 2-hydroxylase (PHYH), the first enzyme of peroxisomal α-oxidation [38]. The somatic mutations in IDH1 were observed in patients with acute myeloid leukemia (AML) [39,40,41] and are possibly associated with a higher proportion of Treg cells in bone marrow [42]. However, the link between the specific peroxisomal localization and role(s) of IDH1 in AML has not been established up to now. In another study, catalase activity was correlated to growth activation in human promonocytic and human B cell lines [43,44]. Catalase is the most highly expressed peroxisomal antioxidant enzyme and acts to prevent oxidative stress by breaking down hydrogen peroxide into oxygen and water. Interestingly, targeting catalase to mitochondria in a mouse model of ataxia-telangiectasia decreases the propensity to develop thymic lymphoma, alleviates bone marrow hematopoiesis and macrophage differentiation in vitro, and leads to the partial recovery of memory T-cell developmental defects [45]. This suggests that the catalase-dependent reduction of mitochondrial ROS controls immune cell proliferation. Similarly, the inhibition of the free radical scavenger Cu, Zn superoxide dismutase (SOD1), which is co-imported into mammalian peroxisomes with a copper chaperone [46], led to cell proliferation arrest and the promotion of cell death in myeloid leukemia cells [47]. Nevertheless, SOD1 is mainly cytosolic and just 0.15% localized inside the peroxisome [46] and there is no evidence for a specific role of peroxisomal SOD1 in this pathology. Of note, the mutation of SOD1 was found to be associated with proliferation of CD11b myeloid precursors in the central nervous system [48].

Among the peroxisomal proteins associated with inflammation listed in Table 1, many are associated with lipid metabolism, especially with VLCFAs which accumulate in case of the peroxisomal β-oxidation defect. X-linked adrenoleukodystrophy (X-ALD) is the most common peroxisomal disorder. This severe and complex neurodegenerative disease is caused by mutations in the *ABCD1* gene. The latter encodes for a peroxisomal transporter of VLCFAs, allowing their entry into the peroxisomal β-oxidation pathway [49]. Early investigation on mononuclear cells in the brain and cerebrospinal fluid of patients developing X-ALD reported that several immune cell types such as T helper and T cytotoxic lymphocytes, B cell subtypes, and monocytes/macrophages mobilize to the brain lesions [50]. However, it has not been established whether the observed phenotype was caused by dysfunctional activities of peroxisomes in the immune cells themselves. Recent reports raised the hypothesis that failures in peroxisome core metabolic pathways, such as β-oxidation of VLCFAs, in immune cells lead to neuronal inflammation, which might be the major cause of neurodegenerative defects observed in X-ALD. Peroxisomal ABC transporters were found differentially expressed in the major CD34^+^-derived immune cell types [51]. ABCD1 and ABCD2, which present a partial functional redundancy for saturated and monounsaturated VLCFAs, show a mirror expression profile between monocytes, B cells, and T cells. In monocytes, ABCD1 is highly expressed, while ABCD2 is extremely low. The B cells show an intermediary level of both ABCD1 and ABCD2 and in T cells, the level of ABCD1 is very low and ABCD2 is higher. Of note, the *ABCD3* gene, which is associated with peroxisomal transport of medium-, long-, branched-chain fatty acids, and bile acid precursors [52,53], is equally expressed in the above described cells. Interestingly, in B cells and in T cells, the ABCD1 deficiency is compensated by the higher expression of ABCD2 [51]. Consistently, a higher accumulation of VLCFA was shown in *Abcd1/Abcd2* double-deficient mouse peritoneal macrophage, compared to the single *Abcd1* deficiency [54]. The detailed consequences of peroxisomal defects in lymphocytes have not been investigated yet. However, in phagocytes (macrophages, microglia cells), the ABCD1 deficiency and the failure to transport VLCFA-CoAs into the peroxisomal matrix lead to lipid modifications concomitant with proinflammatory skewing [55,56]. The up-regulation of pro-inflammatory genes as well as *Trem2* (triggering receptor expressed on myeloid cells 2), which is involved in microglial polarization and phagocytosis control, was also observed in CRISPR/cas9-induced acyl-CoA oxidase 1 (ACOX1)-deficient BV-2 microglial cells [57]. ACOX1 is the first and key enzyme of peroxisomal β-oxidation and provides trans-2-enoyl-CoAs substrates to multifunctional protein-2 (MFP2), the second enzyme of the pathway. Similarly, a MFP2 deficiency in mouse brain microglia provokes intrinsic dysregulation of their pro-inflammatory and proliferative phenotypes but preserves their appropriate responses to inflammatory stimuli [58].

## 3. Peroxisomes Orchestrate the Immune Response Strategies during Systemic Infection

**Figure 1:**
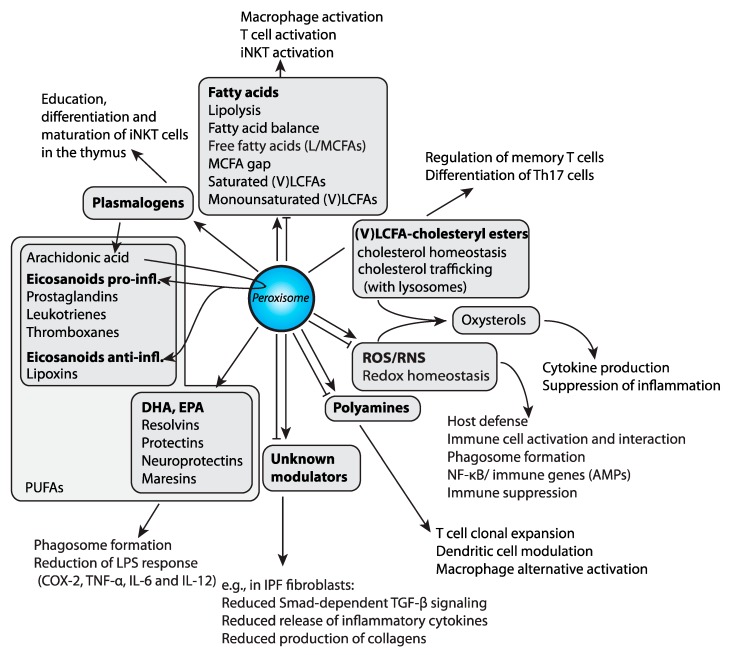
Peroxisomal metabolites and immunity. The metabolites influenced by peroxisomal function (in bold) with downstream processes or derived metabolites (listed in grey boxes) with examples of their roles in the immune system activation and regulation are depicted. Peroxisomal β-oxidation is both involved in the degradation and synthesis of polyunsaturated fatty acids (PUFAs). Moreover, peroxisomes are needed for the production of plasmalogens (ether lipids), which in turn are a source for PUFAs. Arachidonic acid is used for the production of eicosanoids with proinflammatory (prostaglandins, leukotrienes, thromboxanes) or anti-inflammatory functions (lipoxins). Docosahexaenoic acid (DHA) and eicosapentaenoic acid (EPA) serve as precursors for resolvins, protectins, and maresins, which are important for the resolution of inflammation. Furthermore, peroxisomes are needed for the regulation of overall fatty acid composition by degrading very long-chain fatty acids (VLCFAs) and regulating the catabolism of LCFAs/MCFAs. Additionally, peroxisomes are involved in cholesterol homeostasis, intracellular trafficking of cholesterol, and the balance of free and esterified cholesterol. Oxysterols, formed through enzymatic or non-enzymatic cholesterol oxidation, are impacted by the redox balance in the cell, which depends on peroxisomal function. Peroxisomes are involved in the degradation and production of polyamines and might play a role in the degradation and production of other yet unknown modulators of the immune system.

The mechanisms by which peroxisomes and peroxisomal proteins might control immune cell activities have recently started to be dissected by multiple studies carried out in different model systems (Figure 1).

**Figure 2:**
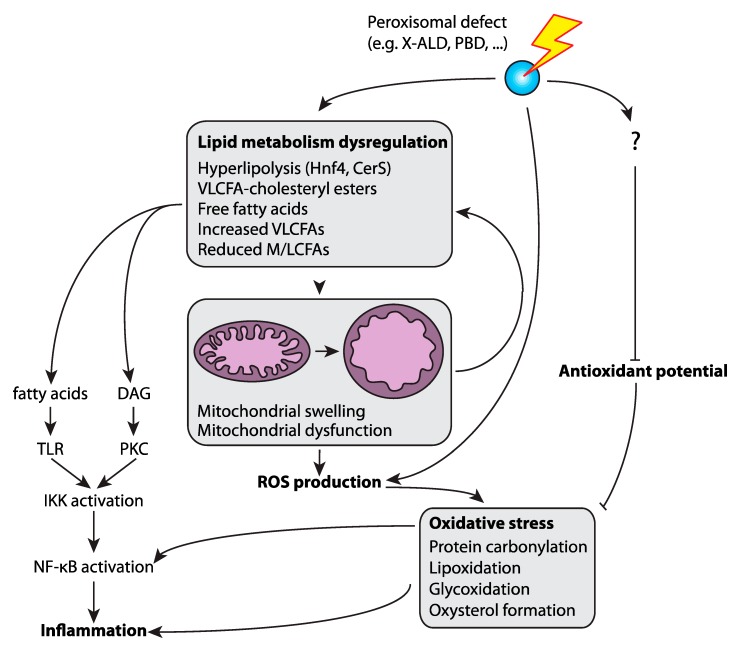
Peroxisomes, oxidative stress and inflammation. Oxidative stress can be the result of increased reactive oxygen species (ROS) production or a decreased antioxidant potential. The defective peroxisomes increase ROS production and reduce the antioxidant ability of the cell. The dysfunctional peroxisomes result also in overall lipid alterations, like the accumulation of VLCFAs and VLCFA-cholesteryl esters. In Drosophila tissue with no functional peroxisomes, the accumulation of free fatty acids was observed. These lipid metabolic defects contribute to mitochondrial damage and swelling, leading to increased ROS production which in turn causes protein carbonylation, lipoxidation, glycoxidation and oxysterol formation, inducing stress pathways and inflammation. Lipid metabolic dysregulation in mammalian systems also causes inflammation via the induction of Toll like receptor (TLR) and Protein kinase C (PKC)-mediated NF-κB signaling.

In *Drosophila melanogaster*, peroxisomal metabolites have been found to modulate phagocytosis (see section below) and the mitogen-activated protein kinases (MAPK) and nuclear factor kappa-light-chain-enhancer of activated B cells (NF-κB) mediated immune responses upon bacteria and fungi infections [27]. *Drosophila* macrophage-like cell lines with no functional peroxisomes, generated by RNA interference-mediated depletion of the peroxisome biogenesis factors (peroxins) Pex5 or Pex7 (designated as PEx5-i or Pex7-i, respectively), had an impaired ROS and RNS response to bacterial infection. ROS and RNS are permeable and diffusible molecules involved in inter- and intracellular signaling during host defense in humans [63,64] and *Drosophila* [65,66,67]. Di Cara et al. showed that the changes in ROS and RNS homeostasis in Pex5-i and Pex7-i immune cells implicate peroxisomes in the host response to infection through their regulation of the signaling that governs the actin network assembly to mediate phagosome formation and pathogen digestions (Figure 1) [27]. Additionally, peroxisome-derived lipids such as DHA also control membrane signaling events that regulate the phagosome actin assembly (Figure 1). Finally, they found that peroxisome metabolism of ROS and RNS control the expression of immune genes during microbial challenge in *Drosophila*. The deregulation of ROS and RNS homeostasis caused by dysfunctional peroxisomes in *Drosophila* macrophages affects the activation of the tumor necrosis factor (TNF) pathway and the MAPK cascade and ultimately inhibit NF-κB nuclear translocation. This leads to the activation of antimicrobial peptides, a response that is a common anti-microbial strategy in *Drosophila* and humans (Figure 1). These defects, as a result of peroxisomal dysfunction, dampen the immune response in the animals and their ability to survive an infection. The requirement of peroxisomes for the regulation of the macrophages’ response to microbes are conserved from *Drosophila* to mammals [27]. Further contribution to inflammation by ROS could arise from mitochondria, which are hyperactive and produce elevated levels of superoxide anions in a *Drosophila* Pex19 mutant [68]. Peroxisome deficiency is also concomitant with an increase in free fatty acids, which are another driver of inflammation [68,69,70]. While associated with the induction of Hnf4 (Hepatocyte nuclear factor 4), the dysregulated expression of acid lipase expression was identified as a main contributor to mitochondrial damage and swelling due to the high amounts of free fatty acids released from triacylglycerol fat stores, which ultimately triggers the production of high amounts of superoxide anions in mitochondria (Figure 2).

In mammals, Cader et al. identified that laccase domain-containing 1 protein (also named FAMIN and encoded by the *Lacc1* gene) localizes to peroxisomes in THP-1 macrophages and regulates fatty acid oxidation of endogenously synthesized lipids [66,67] (Table 1). Interestingly, FAMIN was shown to control ROS production, inflammasome activity and anti-bacterial responses. Of note, the peroxisomal localization of FAMIN has been recently questioned [68]. In another study, Vijayan et al. interrogated the immunomodulatory properties of peroxisomes in mouse macrophages [30]. In RAW 264.7 murine macrophage cell lines and in primary alveolar and peritoneal murine macrophages, the induction of peroxisome proliferation by treatments with 4-phenyl butyric acid, a non-canonical peroxisome proliferator, can reduce the expression of lipopolysaccharide (LPS)-induced pro-inflammatory proteins such as cyclooxygenase (COX-2), tumor necrosis factor alpha (TNF-α), IL-6 and IL-12 two days after stimulation. The RAW cells with no functional peroxisomes due to a mutation in the peroxisome biogenesis factor *Pex14* did not show this reduction in COX2 or any other inflammatory cytokines. The anti-inflammatory effect operated by peroxisomes is dependent on peroxisomal β-oxidation activity, since the deletions of key peroxisomal β-oxidation enzymes cause hyperexpression of COX2 and TNF-α proteins. The authors also suggest that the peroxisomal product necessary to the anti-inflammatory effect in LPS-stimulated macrophages is DHA, a precursor of many lipid resolution mediators (Figure 1), leaving to speculate that peroxisomes produce biolipids to initiate the resolution of inflammation. Interestingly, in this process the activity of NF-κB is not affected, suggesting that peroxisomes can regulate the immune cell activation with different strategies that are NF-κB dependent or independent. Of note, DHA and EPA, but DHA to a lesser extent than EPA, were shown to display resolving effects in human primary monocytes and T-helper lymphocytes [70]. The mechanisms involved in such anti-inflammatory effects could be related to the stimulation of the omega-3 fatty acid receptor G protein-coupled receptor 120 (GPR 120), to biophysical alterations of the cell membranes after incorporation of DHA and/or EPA into phospholipids, to these lipids’ ability to activate nuclear receptors, or to their propensity to act as precursors for various lipid mediators of inflammation (resolvins, maresins, and protectins) [19,71]. The anti-inflammatory effects of omega-3 PUFAs were also confirmed in microglia, although some variability in the secretion of inflammatory cytokines in response to LPS was observed [72,73,74]. Taken together, the central role of peroxisomes in the metabolism of PUFAs definitively establishes this organelle as an actor in the control of inflammation.

The inflammatory and anti-inflammatory properties of peroxisomes have also been associated with the production of ether lipids (see also previous section), exclusively produced by peroxisomes in mammals. Ether lipids are particularly abundant in white blood cells, where in macrophages and neutrophils they represent up to 46% of the total phospholipids [75] with particularly high levels of arachidonic acid in the ether phospholipids. Therefore, in white blood cells, ether phospholipids are a source of arachidonic acid, which is a precursor for the pro-inflammatory eicosanoids [76]. The importance of peroxisomal ether lipids was also highlighted by the recent discovery by Facciotti et al. who described the requirement of ether lipids for the education, differentiation and maturation of iNKT cells in the thymus. The development and maturation of semi-invariant natural killer T cells relies on the recognition of self-antigens presented by CD1d restriction molecules in the thymus. The authors found through the isolation of lipids from thymocytes that ether-bonded mono-alkyl glycerophosphates and their precursors and degradation products stimulate iNKT cells. Mice deficient in the peroxisomal enzyme glyceronephosphate O-acyltransferase (GNPAT), essential for the synthesis of ether lipids, showed a significant alteration of the thymic maturation of iNKT cells and fewer iNKT cells in both the thymus and peripheral organs, which confirmed the role of ether-bonded lipids as iNKT cell antigens. Thus, peroxisome-derived lipids are non-redundant self-antigens required for the generation of a full iNKT cell repertoire [32] (Figure 1).

Fatty acids and reactive species are core metabolites that define various immune functions. However, several peripheral metabolites and pathways that are linked to the core ones shape the complexity of immune cell development, activation and inhibition [77], and some of these rely, at least in part, on peroxisomal metabolism. For instance, polyamines, which are catabolized by peroxisomes [16], have been reported to be important for T cell clonal expansion, and for macrophage alternative activation and dendritic cell modulation [77]. Likewise, cholesterol cellular homeostasis requires peroxisome activity [56,78]. Cholesterol is important to regulate the activity of memory T cells or the differentiation of the Th17 T helper cells, while the cholesterol metabolite 25-hydroxycholesterol instead inhibits and/or promotes inflammatory pathways [77,79]. In addition, the plasma levels of cholesterol and its peroxidative products such as 7-ketocholesterol and 7β-hydroxycholesterol were found increased in X-ALD patients, and oxysterols likely participate in age related diseases [80,81] (Figure 1).

Another connection of the intersection between peroxisome activity and inflammation originated from the studies reported by Oruqaj et al. who demonstrated the implication of peroxisomes in regulating inflammatory signaling not only in immune cells, but also in other tissues such as fibroblasts from human idiopathic pulmonary fibrosis (IPF) [82]. IPF lungs exhibited a significant down-regulation of the peroxisomal biogenesis gene *PEX13* and metabolic genes (e.g., *ACOX1*), and their induction appeared to be regulated by the transforming growth factor-beta (TGF-β) signaling. Furthermore, in the vitro treatment of IPF fibroblasts with the pro-fibrotic factors TGF-β1 or TNF-α was found to down-regulate peroxisomes via the Activator protein 1 (AP-1) signaling pathway. The direct down-regulation of the gene encoding for the peroxisomal biogenesis factor *PEX13* by RNA interference induced the activation of Smad-dependent TGF-β signaling accompanied by increased ROS production and resulted in the release of inflammatory cytokines (e.g., IL-6, TGF-β) and excessive production of collagen I and III (Figure 1). In contrast, the treatment of fibroblasts with ciprofibrate or WY14643, PPARα activators, led to peroxisome proliferation and reduced TGF-β–induced myofibroblast differentiation and collagen protein accumulation in IPF cells. Thus, peroxisomes regulate inflammatory signaling in different tissues.

## 4. Peroxisome Metabolism Protects from Neuroinflammation

Inflammation involves cells, vessels, changes in the extracellular matrix, and many chemical mediators that can be pro- or anti-inflammatory and can modify or maintain the inflammatory response. Neuroinflammation concerns brain, neurons and immune cells, mainly microglial cells, which, in the case of disruption of the blood-brain barrier, can be accompanied by infiltrating cells (lymphocytes, granulocytes and monocytes (macrophages at the tissue level)). Among the proteins that drive inflammation, cytokines are secreted early by the immune system cells and vascular endothelial cells. In X-ALD, the inflammation of the brain is characterized by perivascular infiltration of CD8+ and CD4+ lymphocytes, a decrease in the B cell number, rare plasma cells and the presence of macrophages near areas where myelin is damaged [50,83]. The activated microglia and hypertrophic astrocytes are also found in these demyelinated areas. These lymphocytes, macrophages, microglia, and astrocytes are able to present lipid antigens to immunocompetent cells and trigger an inflammatory response via the expression of the CD1 antigen-presenting molecule [83]. The transcriptome analysis of adrenomyeloneuropathy (AMN) monocytes confirmed a pro-inflammatory status of these cells [55]. In this study, the intrinsic pre-activation profile (due to ABCD1 deficiency), as well as the increased propensity for complete pro-inflammatory activation in response to LPS observed in vitro was reflected in vivo by an increased monocyte/macrophage recruitment in perivascular cuffs at myelopathy sites in the AMN spinal cord. The monocytes showed an increased propensity to migrate to the central nervous system (CNS) lesion sites and, possibly, to cross the blood-brain barrier. However, no significant change was observed in the viability and intracellular redox status. This pro-inflammatory profile observed in monocytes persisted after differentiation into macrophages. In post-mortem samples of young X-ALD patients, adult X-ALD patients, and AMN patients, IL-1 and ICAM-1 were detected in microvessels and astrocytes, while TNF-α was observed in macrophages and more significantly in astrocytes [84]. The accumulation of these cytokines and adhesion molecules overlapped with the infiltration of T lymphocytes (around the vessels of the lesion) [84]. The TNF-α bioactivity in the serum of some patients (e.g., childhood cerebral ALD patients (cALD)) has been suggested as the triggering factor of the activation of the inflammatory status in monocytes [85]. However, in a follow-up study, the same team suggested that an increase in mRNA and protein levels of γ-interferon, IL-1α, IL-2 and IL-6, GM-CSF, TNF-α, chemokines and chemokine receptors in chronic and acute lesions act as potential triggers of the inflammatory status [86]. X-ALD patients (asymptomatic or symptomatic patients with cerebral demyelination (childhood cALD and AMN patients with or without abnormal MRI)) were followed for two to five years to monitor their TNF-α levels in the blood [87]. TNF-α levels were correlated with the progression of demyelination. Following stimulation by LPS, the peripheral blood mononuclear cells (PBMCs) in symptomatic X-ALD patients produced higher levels of TNF-α than the controls. The production of other cytokines did not change between the X-ALD patients and healthy control groups. In patients who underwent bone marrow transplantation, LPS-stimulated PBMCs had the same TNF-α production like the control individuals, even 5 to 8 years after transplantation. Therefore, it seems clear that X-ALD patient monocytes have defects in the TNF-α pathway. In addition, PBMCs from 17 patients (asymptomatic, AMN and cALD subjects) stimulated by LPS and tested for cytokine production showed increased production of IL-12 and TNF-α [88]. The patients’ PBMCs are able to induce a Th1-type response to a stimulus [88]. The postmortem immunohistological analysis carried out on the brain of children affected by cALD showed an induction of proinflammatory cytokine (e.g., IL-1α, TNF-α, IL-6 and GM-CSF) and chemokine mRNAs (e.g., CCL2 (MCP-1), CCL4 (MIP-1β) and CCL7 (MCP3)) in demyelinating lesions [89]. In the inflammatory area of cALD brains (area surrounding the plaque in the white matter), the increased expression of inflammatory genes was correlated with the presence of VLCFAs and cholesteryl ester in the plaque, as well as a loss in all of the above-mentioned areas of cholesterol and sphingomyelin [89].

When the presence of pro- and anti-inflammatory cytokines was measured in the plasma of three different X-ALD phenotypes (asymptomatic, AMN and cALD), a correlation between their concentrations and VLCFA levels was found [90]. Interestingly, also asymptomatic patients have higher plasma levels of pro-inflammatory cytokines (Il-1β, IL-2, IL-8 and TNF-α) compared to the control group [90]. On the other hand, anti-inflammatory cytokines, such as IL-4, were found in AMN and asymptomatic patients, while the IL-10 concentration was significantly higher in asymptomatic patients than AMN, cALD, and the controls [90]. Only AMN patients had very high levels of IL-5. In contrast, Lund et al. did not find an increase in pro-inflammatory cytokines in serum but an increase in IL-8, IL-1ra, MCP-1, and MIP-1β, in cerebrospinal fluid (CSF) from cALD patients [91]. In addition, regarding the effect of inflammation on the extracellular matrix, it has been shown that MMP-10 and TIMP-1 metalloproteinase levels were higher in cerebrospinal fluid in boys with cALD and correlated with the severity of the inflammation [92].

The involvement and presence of inflammatory molecules in disease progression has also been demonstrated in different cellular and animal models with peroxisomal defect. An ACOX1 deficiency is indeed characterized by the activation of the IL-1 pathway, as well as the induction of cytokines IL-6 and IL-8 by the MAPK and p38 MAPKK pathways [93]. In an *ABCD1* deficient mouse model, macrophages secreted higher levels of IFN-γ, TNF-α, IL-6, and IL-12p70 compared to wild type mice [94]. The primary mouse astrocytes silenced for *Abcd1* and/or *Abcd2* show an inflammatory response (increase in pro-inflammatory cytokines: TNF-α, IL-1β) characteristic of human cALD, which is mediated by the transcription factors NF-κB, AP-1 and C/EBP [95]. Further, 25-hydroxycholesterol has been identified as a contributor to brain inflammation via the activation of the NLRP3 inflammasome (nucleotide-binding oligomerization domain-like (NOD like) receptor containing pyrin domain 3) [79].

The potential relationship between inflammation, peroxisomal abnormalities, and oxidative stress came from observations in patients (or respective mouse models) affected by X-ALD [96] (Figure 2). An unbalanced ROS status in the central nervous system (CNS) has been linked directly to several key features of neurodegenerative diseases, such as a high level of inflammation, apoptosis of a subset of neurons and glia, and an increased number of microglia in the brain area. Furthermore, the activation of resident macrophages by high levels of ROS further exacerbates these disorders by inducing additional ROS and inflammation. The process results in a positive feedback loop, which can become self-sustaining and lead to systemic brain dysfunction. In demyelinating lesions of cALD brains, the induction of proinflammatory cytokines has been paralleled by the upregulation of the *NOS2* gene that encodes for the inducible nitric oxide synthase (iNOS) [89]. Moreover, lymphocytes from X-ALD patients exhibit higher production of NO and cytokines, such as TNF-α and IL-1β, together with overproduction of ROS and the accumulation of the amount of NADPH oxidase gp91 (PHOX) subunit protein, an integral component of the nicotinamide adenine dinucleotide phosphate (NADPH) oxidase complex that generates reactive oxygen species in circulating phagocytes and microglia cells [97,98]. Interestingly, fibroblasts derived from X-ALD patients also showed an overproduction of ROS that led to protein carbonylation, lipoxidation and glycoxidation as indicated by the increased levels of glutamic semialdehyde (GSA), aminoadipic semialdehyde (AASA), N1-carboxyethyl-lyine (CEL) and N1-malonedialdehyde-lysine (MDAL), respectively [99,100]. In the *Abcd1* knockout mice, which develop a spinal cord disease mimicking adrenomyeloneuropathy (AMN) in adult patients, MDAL accumulates in the spinal cord of *Abcd1* knockout mice in consequence of lipoxidative damage to proteins. In these mice the GSA, AASA and CEL levels were also increased in the spinal cord while the expression of antioxidant enzymes such as catalase, glutathione peroxidases (e.g., GPx1, GPx3, GPx4) and superoxide dismutases (i.e., SOD1, SOD2) were downregulated in both the cortex and the spinal cord [99]. Further studies carried out in X-ALD patient fibroblasts demonstrated that the overproduction of ROS might, at least partly, be caused by mitochondrial dysfunction. In these studies, the accumulation of the saturated unbranched VLCFAs species, C26:0, induced oxidative stress and unpaired oxidative phosphorylation, triggering ROS production from the electron transport chain complexes. The excessive ROS production was associated with a malfunction of the mitochondrial complex V oxidative phosphorylation activity, as shown by the high-resolution respirometry on spinal cord slices from an *Abcd1* deficient mice [101]. Moreover, it was also suggested that defects in the activity of key mitochondrial trichloroacetic acid (TCA) cycle enzymes, such as pyruvate and α-ketoglutarate dehydrogenases and aconitase, might be an important pathophysiological factor in neurodegenerative diseases, by causing mitochondrial dysfunction and bioenergetic failure [102] (Figure 2). The transcriptomic analysis on spinal cords extracted from *Abcd1* deficient mice, and from normal-appearing white matter of AMN and CALD patients, suggested the existence of a link between inflammation and the ROS overproduction in mitochondria [103].

Various in vivo and in vitro studies show evidence that oxidative damage occurs in neurons of X-ALD patients. Furthermore, the administration of antioxidant combination therapies slows down clinical progression and reverses axonal damage in a mouse model of X-ALD. These data support a model that oxidative injury is a main contributing etiopathogenic factor in this disease. Given the role that peroxisomes play in oxidative stress, there is growing evidence that suggest that peroxisomes have similar contributions to pathophysiological features in other neurodegenerative diseases such as Alzheimer’s disease, multiple sclerosis, Parkinson’s disease, and Huntington’s disease, in which high levels of inflammatory cytokines associated with oxidative stress have been detected [104]. In some of them, such as Alzheimer’s disease and multiple sclerosis, peroxisomal dysfunctions were observed [105,106,107], thus consolidating the link between peroxisomal dysfunctions, oxidative stress and inflammation in neurodegeneration.

## 5. Peroxisome Metabolism during Aging

Low-grade chronic inflammation is a pervasive age factor associated with age-related complications such as diabetes, cancer, and neurodegeneration. The aging of cells is concomitant with increased ROS levels, and this is, at least in part, due to increased peroxisomal biogenesis [108] and decreased catalase activity [109]. Mitochondrial fragmentation and an increase in mitochondrial ROS production have been suggested as key drivers of aging. However, mitochondrial and peroxisomal fission as well as ROS production are interconnected, and it became evident in recent years that peroxisomes are major contributors to the aging process. The importance of peroxisomal function in cellular and organismal aging is conserved from single-cell organisms like yeast, to complex invertebrates like *D. melanogaster*, to higher vertebrates like humans. As amply described in the previous sections, peroxisomal metabolism contributes to the regulation of inflammation. Their ROS-scavenging capacity is probably one of the main mechanisms involved in such control. Interestingly, like mitochondria, the alteration of organelle dynamics is sufficient to alter SOD2 and catalase expression and increase oxidative stress [110].

**Figure 3:**
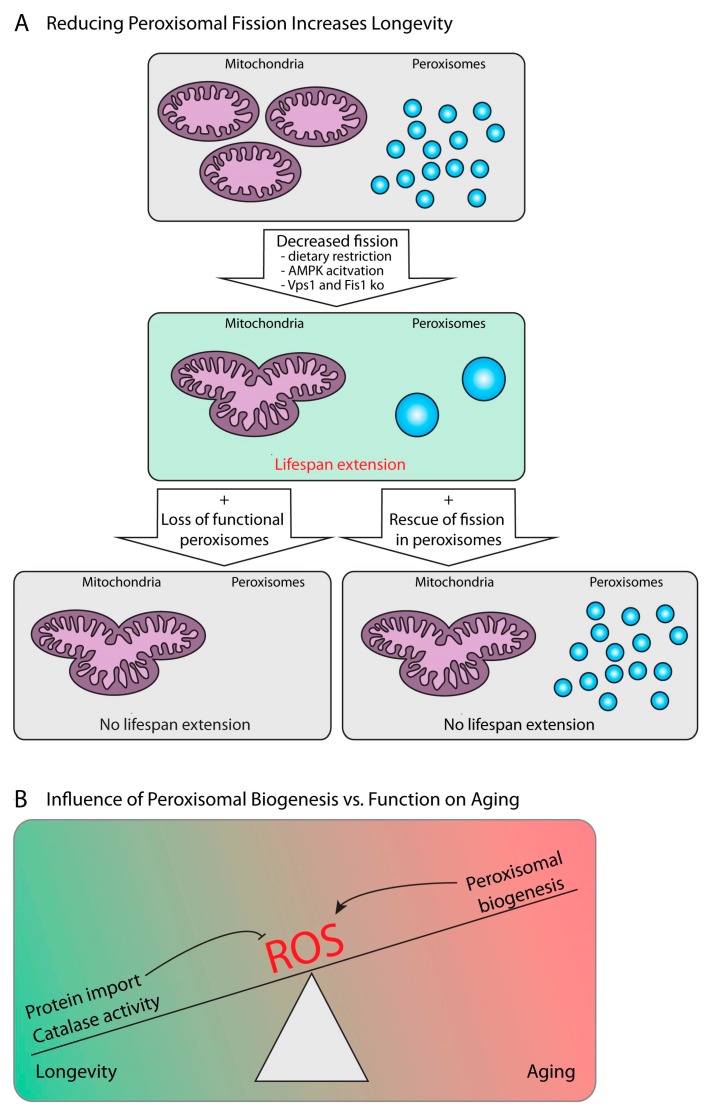
Peroxisomes and aging. (**A**) Mitochondria undergo cycles of fusion and fission, resulting in mitochondrial networks or fragmented mitochondria, respectively. If fission is decreased and mitochondria form a network (e.g., as a result of dietary restriction or AMPK activation, or mutation of Vps1 and Fis1 in yeast), lifespan increases. However, when the mitochondrial fission machinery is reintroduced, but targeted exclusively to peroxisomes (e.g., by overexpression of a Pex15-Fis1 fusion protein, lower right panel), or when peroxisomal functions are missing altogether (e.g., by additional mutation of the peroxisomal biogenesis factor Pex5, lower left panel), lifespan extension is lost, even though mitochondria are still forming a network. (**B**) A summary of the current understanding of the effect of peroxisome activity on aging. Peroxisomes’ effect on aging is linked to the production of reactive oxygen species (ROS): The proper activity of the peroxisomal ROS scavenging enzyme catalase is essential to promote longevity by reducing oxidative stress, while overall peroxisomal abundance (peroxisomal biogenesis) increases oxidative stress and therefore promotes aging.

Mitochondrial function is closely connected to the aging process (reviewed in [111]). However, mitochondria and peroxisomes cooperate closely and share the machinery needed for their fission. Therefore, some of the effects relevant for the aging process attributed previously to mitochondria are in addition or exclusively caused by peroxisomes. For example, in aging *Saccharomyces cerevisiae* cells, mitochondria are fragmented, and the reduction of the mitochondrial fission machinery by the deletion of genes encoding for Dnm1, Fis1 or Mdv1 prolongs the lifespan. Thus, mitochondrial fragmentation has been suggested to induce cell aging and death [112]. However, since these genetic manipulations likely also affect peroxisomes as the fission machinery is shared between mitochondria and peroxisomes, it requires directed experiments to distinguish peroxisomal from mitochondrial contribution to the aging process. Lefevre et al. showed that fission was blocked in both mitochondria and peroxisomes in Vps1/Fis1 double mutant yeast cells, while rescued with Fis1 restored organelle morphology. In contrast, yeast cells expressing a Fis1-Pex15 fusion construct in a Vps1/Fis1 mutant background showed a selective block of mitochondrial fission while leaving peroxisome fission unaffected, since Pex15 restores the recruitment of the fission machinery to the peroxisome. With that, the authors show that lifespan extension due to Fis1 deletion is caused by a block in peroxisomal fission rather than mitochondrial fission [113] (Figure 3A).

Another example is the role of mitochondria and peroxisomes in nutrient dependent aging effects. The nematode *Caenorhabditis elegans* has a long history in longevity and aging studies. It is well established that dietary restriction extends lifespan, and genetic manipulation of nutrient signaling pathway components also impacts on lifespan. One of the proposed modulators of dietary restriction effects on lifespan is the AMP-activated protein kinase (AMPK) (reviewed in [114]). In 2017, Weir and colleagues showed that worms expressing a constitutively active version of AMPK had an extended lifespan and maintained a mitochondrial network. The longevity effect was dependent on mitochondrial fusion, since the mutation of the fusion factor Fzo1 suppressed lifespan extension caused by constitutive AMPK activity or by dietary restriction. Similarly, worms deficient for both fusion and fission (Drp1/Fzo1 double mutants) maintained balanced mitochondrial network homeostasis and lived longer. However, this effect depends on peroxisomal function, since lifespan extension was blocked when Pex5 was knocked down in the mutant worms. Indeed, peroxisomes were larger and less fragmented in Drp1/Fzo1 double mutants than in wildtype worms, since the shared fission machinery was not working (Figure 3A). Finally, peroxisomal function is also required for both AMPK- and dietary restriction-mediated lifespan extension [115].

In the fruit fly *D. melanogaster*, the transcript levels of peroxisomal genes show age-dependent enrichment [116], but whether their number or fission state alters with age is not characterized yet. A cross-species study from Zhou and colleagues showed that the expression of genes for lipid metabolism and peroxisome biogenesis were anticorrelated with lifespan in mice subjected to different dietary interventions which affect longevity [108]. In the same line of evidence, they showed lifespan increase in fly models with a decreased expression of the peroxisome assembly factors Pex1 and Pex13 and a *C. elegans* model with Pex13 knock-down. They could attribute this lifespan extension to decreased ROS levels. Their results therefore suggested that peroxisome biogenesis is negatively correlated with lifespan, but they also provided evidence that mitochondrial biogenesis positively correlates with lifespan, and that biogenesis of the two organelles competes during aging [108] (Figure 3B). Thus, during aging, the expression of genes for peroxisomal biogenesis and lipid metabolism increases [108], while protein levels for factors involved in matrix protein import, but also catabolic processes, decrease [117], leading to an imbalance in peroxisomal activity and abundance and its ROS-scavenging capacity (Figure 3B). Furthermore, it has been demonstrated that mouse life span is extended by overexpressing and targeting catalase into mitochondria [118]. In contrast to glutathione peroxidase, targeting catalase to mitochondria allows the degradation of H_2_O_2_ independent of the availability of electron donors such as NAD(P)H/H^+^. Importantly, the results on the overexpression of the ROS-scavenging enzymes catalase and superoxide dismutase in extending the lifespan of flies [119,120] is still controversial, since ectopic expression of catalase in the mitochondria of *D. melanogaster* was shown to enhance stress resistance but not longevity [121]. Thus, more research is required to determine the importance of peroxisome abundance, activity and turnover (i.e., removal of old peroxisomes) for aging.

The reduced import of peroxisomal proteins as a factor in aging was also analyzed in an important study in human fibroblasts [109]. The aging of fibroblasts is characterized by a decrease in the import efficiency of PTS1-containing proteins, especially catalase. This decrease becomes evident already before the cells reach senescence. Catalase is particularly affected because it has a non-canonical PTS1 motif, KANL, in contrast to the common SKL. KANL has a lower affinity to the import receptor Pex5 and is thus prone to age-related lower import efficiency [109]. This shows that, across different species, peroxisomal ROS production is a key driver of aging.

Given the importance of peroxisomal lipid synthesis in the production of lipid classes essential for neuronal development and maintenance affecting neuronal aging, for example plasmalogens, it is likely that peroxisomes are also associated with age-related neuropathies like Alzheimer’s disease (AD). The unique substrates of peroxisomal fatty acid oxidation, very-long-chain fatty acids (VLCFA), accumulate in the brains of AD patients, and the amount of VLCFA correlates with the formation of neurofibrillary tangles. Further, the decrease of plasmalogens observed in the gyrus frontalis of AD patients correlates with the formation of neurofibrillary tangles. The severity of AD pathology is connected to peroxisome abundance [105], which correlates with pro-aging effects of peroxisomal biogenesis in mice and human fibroblasts. This shows that both the function of peroxisomes to maintain ROS equilibrium, as well as their role in lipid anabolism, are determinants of healthy aging (Figure 3B).

## 6. Peroxisomes Act as Signaling Platforms to Activate Antiviral Responses

**Figure 4:**
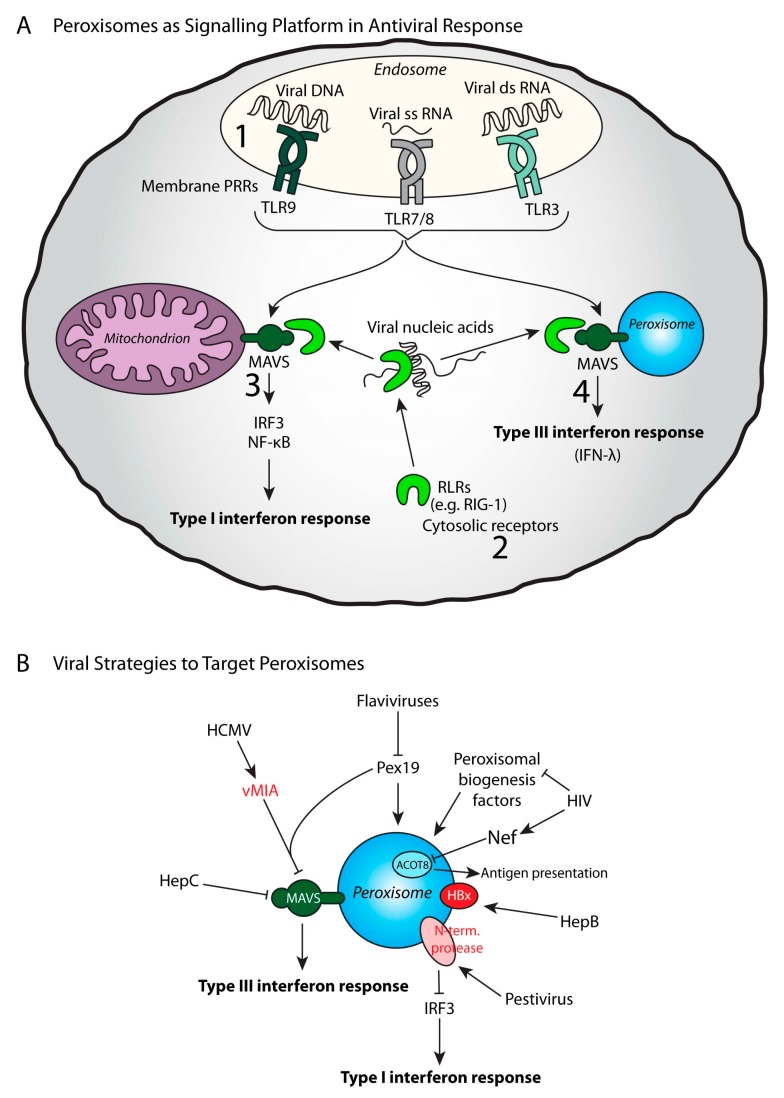
Peroxisomes in an antiviral response. (**A**) Peroxisomes are signaling hubs for the induction of a Type III interferon response by harboring the antiviral signaling protein MAVS. Viral nucleic acids are recognized (1) by the membrane receptors like the Toll like receptors (TLRs) 3, 7, 8 and 9, or by (2) cytosolic receptors (RIG-1 like receptors, RLR). RLRs activate mitochondrial antiviral signaling adaptor (MAVS) to defeat viruses. The location of MAVS on either mitochondria or peroxisomes leads to different responses: (3) mitochondrial MAVS responds via IRF3 and NF-κB signaling, leading to initiation of Type I interferon response, while (4) peroxisomal MAVS can initiate a Type III interferon response. (**B**) Some viruses utilize strategies to target peroxisomes in order to circumvent the immune response. Hepatitis C (HepC) and Human cytomegalovirus (HCMV) target the MAVS function, thereby inhibiting the Type III interferon response. HCMV viral protein vMIA interacts with Pex19 in order to target MAVS. Certain flaviviruses and HIV target peroxisomal biogenesis factors, thereby inhibiting overall peroxisomal functions. In addition, the HIV protein Nef interacts with peroxisomal ACOT8, which interferes with antigen presentation and T cell activation. Some viral proteins localize to peroxisomes: the pestivirus protein N-terminal protease is located on peroxisomes and targets the Type I interferon response by inhibiting IRF3 function, and the Hepatitis B (HepB) protein HBx localizes to peroxisomes and seems to promote hepatocellular carcinogenesis.

Peroxisomes control pro-inflammatory and immune pathways not only by producing bioactive metabolites important to drive immune signaling, but, like mitochondria, they can recruit signaling proteins to their membrane to promote their activation in response to a stimulus. This strategy is essential to trigger interferon-mediated antiviral signaling (Figure 4A).

A direct link between peroxisomes and viruses was first described in 1983 in plants [122]. A few years later, the replication and assembly of duck hepatitis B virus was suggested to occur in the vicinity of peroxisomes [123]. In mammals, several peroxisomal changes were also described in the liver of mice infected by the hepatitis virus, but it was not clear if these observations were directly linked to viruses or to the hepatic modifications induced by the virus [124,125,126]. Indeed, peroxisomes in mammals were demonstrated to participate in the replication of several viruses, housing several important viral proteins, and also participating in providing lipids essential for virus envelopes such as ether lipids [127]. For instance, peroxisomal biogenesis was shown to be essential for replication of several viruses such as Kaposi’s sarcoma-associated herpesvirus [128], herpes simplex virus, or Human cytomegalovirus [129]. In addition, vFLIP, a viral protein essential for HHV-8 latency and associated pathogenesis, was found to be stabilized on peroxisomes [130] (Figure 4B).

**Figure 5:**
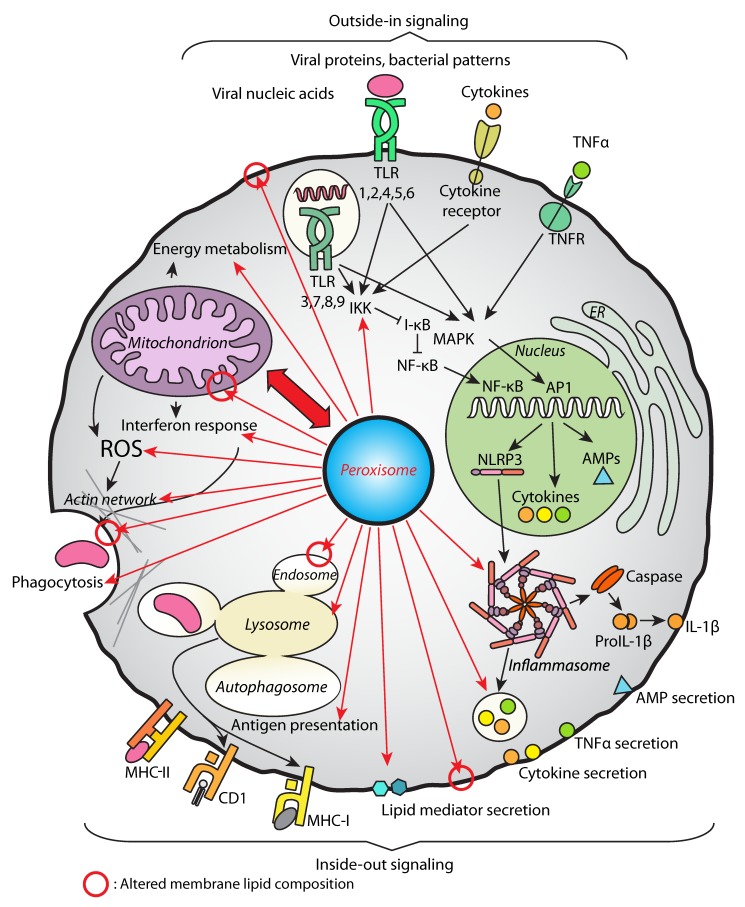
An overview of immune processes regulated by peroxisomes. The overall lipid perturbations in membranes due to peroxisomal defects (indicated by red circles) could impact the function of membrane-localized receptors and signaling between the cells (top: outside-in signaling examples including pattern recognition and pro-inflammatory signals, bottom: inside-out signaling examples including antigen presentation and secretion of immune system mediators). The altered composition of membrane lipids also influences membrane properties relevant for vesicular trafficking (phagocytosis, endocytosis, lysosomal function etc.) and functions of the inner and outer mitochondrial membranes. Peroxisomal functions impact on the immune pathways, like NF-κB and MAPK, which regulate the production of immune regulators such as cytokines and antimicrobial peptides (AMPs). While a direct link between peroxisomal defects and NLRP3 activation has not been demonstrated so far, peroxisomes may influence the function of the NLRP3 inflammasome. Peroxisomes contribute to the secretion of immune system mediators (like IL-1β, which is regulated by the inflammasome, but also many other pro- and anti-inflammatory cytokines, including TNFα, and lipid mediators with pro- and anti-inflammatory capacity). Peroxisomes impact on ROS signaling, play a role in phagocytosis regulation (including regulation of the actin cytoskeleton), and in the antiviral interferon-mediated response. Peroxisomes closely cooperate with mitochondria (red double arrow) with respect to membrane contact sites, ROS production, and lipid exchange, thereby influencing energy metabolism and lipid catabolism.

In this peroxisome-virus relationship, the demonstration by Dixit et al. in 2010 that peroxisomes constitute an antiviral signaling platform and thus contribute to innate immunity was a real breakthrough in the peroxisomal field [63]. More recently, a proteomic analysis of peroxisome-enriched fractions from Sendai virus-infected or uninfected HepG2 cells revealed a large number of proteins significantly changed by the viral infection, 25 of which are linked to the immune response [131]. The main antiviral signaling pathways depend on the detection of viral products (proteins, lipids, nucleic acids) by pathogen recognition receptors (PRRs). Viral proteins can be recognized by PRRs such as TLR2/1, TLR2/6, and TLR4, while viral DNA, single-stranded RNA, and double-stranded RNA are recognized by TLR9, TLR7/8 and TLR3 respectively (TLR3) [132,133]. Cytosolic receptors such as RIG-I-like receptors (RLRs) are also involved in the recognition of viral nucleic acids [134]. Binding to viral RNA induces conformational changes in RLRs, which allow them to interact with adapter proteins on the surface called mitochondrial antiviral signaling adaptor (MAVS). MAVS is a tail-anchored protein first described on the outer membrane of mitochondria [23]. The downstream signaling pathways activate transcription factors such as NF-κB and interferon regulatory factors, leading to the production of pro-inflammatory cytokines and type I interferons. Actually, MAVS localization was demonstrated to be multiple including mitochondria, a specific region of the ER membrane called mitochondrial-associated ER membrane, and peroxisomes [63,135,136]. The peroxisome-associated type III interferon response led to the hypothesis that the differential localization of MAVS to peroxisomes and mitochondria drives different antiviral signaling programs [63] (Figure 5A). Peroxisomes with their abilities to interact with other organelles and to house MAVS are now considered as an important signaling platform for the antiviral response. RLR-mediated type III interferon expression can be induced by various viruses, including reoviruses, Sendai- and dengue viruses [137]. Recently, SLC15A3, a proton-coupled histidine and di-tripeptide transporter previously found in lysosomes, has been reported to be partially localized in peroxisomes [138]. While known to inhibit chikungunya viral replication, SLC15A3 was demonstrated to inhibit HSV-1 replication and enhance type I and type III interferon responses.

Peroxisomes activate antiviral defense strategies, but they can also be the targets of viruses in order to escape the immune response (Figure 4B). The West Nile and dengue virus (flaviviruses) infection was shown to trigger peroxisomal biogenesis inhibition [139]. This peroxisome loss was associated with capsid protein-dependent sequestration and the degradation of the peroxisomal biogenesis factor PEX19 and would explain why the induction of type III interferon is impaired. HIV infection has also been associated with peroxisomal biogenesis inhibition, but through a different mechanism [140]. In this particular case, the up-regulation of miRNAs that repress peroxisome biogenesis factors was identified. The human cytomegalovirus (HCMV) developed a distinct evasion mechanism involving a virally-encoded protein vMIA, which interacts with PEX19 and targets the peroxisomal MAVS to inhibit the antiviral response. The cleavage and inactivation of both mitochondrial and peroxisomal MAVS resulting in suppressing the activation of the interferon response was also reported in the case of Hepatitis C virus [141,142]. Peroxisomal targeting of the HBx protein of Hepatitis B virus has also been described and has been suggested to increase hepatocellular carcinoma progression [143]. The N-terminal protease of pestivirus was also found to be associated with peroxisomes and inactivates IRF3, one of the main regulators of interferon production [144]. Another example concerns the HIV viral protein Nef, which appears to interact with the peroxisomal thioesterase ACOT8. This interaction is thought to participate in Nef-mediated MHC-I downregulation and the prevention of T-cell activation [145,146,147].

## 7. Peroxisomal Functions are Linked to Phagocytosis Capacity

For decades, phagocytosis was considered a mere immunological process. However, it is now appreciated that phagocytosis also plays a critical role in embryonic and post-natal development, as well as in tissue remodeling and the maintenance of overall homeostasis [148]. Developmentally relevant phagocytosis is essential within the developing CNS for the elimination of excessive unwanted synapses and for the removal of apoptotic neurons and oligodendrocyte progenitor cells that are overproduced. In the adult brain, constant immune surveillance contributes to synaptic transmission and plasticity, to phagocytosis of apoptotic cells and myelin debris, and to maintain tissue homeostasis. Within the aging CNS, the main professional phagocytes, i.e., microglia, become senescent and display impaired myelin debris/apoptotic cell clearance and excessive synapse pruning [149]. Phagocytosis also plays a critical role in neurodegenerative diseases. Indeed, in Alzheimer’s disease and multiple sclerosis, microglia contribute to the pathology, which is in part mediated by phagocytosis of toxic amyloid-beta or by myelin debris originating from the destruction of myelin sheaths, respectively [149]. Of note, peroxisome alterations and VLCFA accumulation were reported in these diseases [105,106]. Microglia is also considered a key contributor to the physiopathology of X–ALD. The benefit of hematopoietic stem cell gene therapy based on the correction of CD34^+^ cells from X-ALD patients with a lentiviral vector encoding ABCD1, is likely due to the replacement of deficient brain microglia to the gene-corrected microglia-like cells [150]. Bergner et al. demonstrated that the immune-phenotype of microglia was altered early in lesion evolution, and that microglia damage preceded the major destruction of oligodendrocytes and myelin [151]. Recent experimental evidence indicated that in X-ALD patients, microglial cells showed defects in phagocytosis that contribute to the neurodegenerative process. Indeed, in AMN patients, the adult form of X-ALD, the spinal cord displayed microglia activation and an increased expression of several phagocytosis-related markers such as Milk Fat Globule-EGF Factor 8 (MFGE8) and TREM2 on viable neurons, which indicates a contribution to neuron degeneration through phagocytosis [152]. Similarly, an activation marker for monocytes/macrophages (MRP14) and a microglia/macrophage phagocytosis marker (CD68) accumulate in demyelinating lesions in AMN spinal cord tissue [55]. The observed impact of peroxisomal defects on the expression of the phagocytosis-related marker TREM2 suggests profound changes in microglial functions related to phagocytosis, as demonstrated by studies of microglia cells mutated for ABCD1 or ACOX1 [56,57].

Phagocytosis is a complex process requiring energy, specific cell surface contacts, membrane invagination, and dedicated internalization processes. It is well known that lipid membrane composition alters phagocytosis, and depending on its lipid environment, microglia likely modifies its function toward clearance. Since peroxisomes are involved in the catabolism of VLCFAs, their dysfunction leads to excess VLCFA incorporation in cell membranes, which could affect the phagocytic abilities of microglia and other phagocytic cells. The membrane properties are also known to be modified due to changes in their cholesterol content. A change in the external milieu of in vitro cultured macrophages, in particular the cholesterol/lipid content, resulted in a change in the ability of macrophages to clear opsonized bacteria [153]. Of note, peroxisomal defects were associated with cholesterol accumulation and altered intracellular transport of cholesterol [154]. It should be noted that the accumulation of cholesteryl esters of VLCFAs, a hallmark of peroxisomal disorders, has also been suggested to trap cholesterol and modify cholesterol levels in membranes. PUFAs and MUFAs, whose metabolism partly depends on peroxisomal β-oxidation, are also described as modulators of phagocytosis in many examples [72]. The process of phagocytosis is intimately related to inflammation, and the incorporation of DHA into the cell membrane alters the composition of lipid rafts, leading to the expulsion of inflammatory receptors such as toll-like receptors TLR2 and TLR4 from lipid rafts, hence inhibiting their activation [155]. DHA and EPA were shown to increase the phagocytosis ability of microglia towards amyloid-β peptide [156]. The treatment with DHA or EPA has been shown to enhance macrophages’ phagocytosis capacity of Zymosan particles [157]. The phagocytosis rate of bacteria by murine macrophage cell line RAW264.7 supplemented with PUFAs was also found increased [158]. Of note, EPA was shown to increase adhesion of bacteria to human monocytes [159]. Phagocytic activity of human monocytes [160] as well as caprine monocytes and neutrophils [161,162] were also demonstrated to be induced by PUFAs. Besides their action on membrane properties, other mechanisms could be involved, since DHA and EPA are also known as signaling molecules and as precursors for pro-resolving factors. Interestingly, the observed stimulation of phagocytosis of the amyloid-β peptide by EPA- or DHA-induced microglia appeared to be biphasic with an immediate activation, followed by a steady-state period and a new stimulation occurring 24 h later [156]. This delayed activation could be linked either to eicosanoids or docosanoids, which have been demonstrated to induce phagocytosis and resolve inflammation [19] or to modulate transcription. DHA and EPA are both PPARγ ligands, and the inhibition of PPARγ was shown to decrease phagocytosis of apoptotic cells in macrophages by inhibiting the expression of cell surface receptors and secreted molecules (CD36, TG2, AXL and PTX3) involved in the engulfment process [163].

In 1979, employing the alkaline diaminobenzidine technique to visualize peroxisomes, Eguchi et al. located peroxisomes in close proximity to phagosomes in peritoneal macrophages during phagocytosis of latex beads [164]. Since cytochemical evidence of catalase discharge from peroxisome to phagosome was observed, the authors suggested that the role of peroxisomes in phagocytic activity is this discharge with the goal to reduce the oxidative burst generated by microbicidal ROS accumulated in the phagosome [164]. The hypothesis is that both mitochondria and peroxisomes control the process. The mitochondria discharge ROS to kill bacteria in the phagosome, while peroxisomes discharge catalase to modulate the damage that the oxidative burst might cause to the cell. Almost 40 years later, performing live imaging analysis of *Drosophila* macrophages, Di Cara et al. demonstrated that peroxisomes move randomly in the cytoplasm of uninfected cells, while they move directionally towards the early phagosome in infected cells [31]. Moreover, the authors reported that peroxisomes are necessary for the formation of the phagosome. Peroxisomes control the process by regulating cellular ROS and RNS, which in turn regulate cellular signaling, mediating the organization of the actin cytoskeleton for the formation of the phagosome [31].

## 8. Conclusion and Future Remarks

The importance of peroxisomes in immunity and inflammation has become clear in the past ten years. Apart from being a central metabolic organelle essential for organism developmental survival, the peroxisome has emerged as a cellular factor that contributes to modulate the immune response and inflammation using various strategies summarized in Figure 5.

Peroxisomes contribute to the compartmentalization of the immune response pathways by operating as signaling platforms to trigger the activation of immune signaling such as MAVS-mediated antiviral responses. Moreover, peroxisomes function as immunometabolic hubs contributing to cellular metabolites such as ROS and unsaturated fatty acids to control the immune cell development and activation, and to modulate inflammatory pathways in different immune cells and tissues. Peroxisomal control of cellular reactive anionic species production and turnover fine-tunes phagocytosis in macrophages and microglia cells, modulates NF-κB activities and regulates the synthesis and/or release of inflammatory cytokines. Additionally, peroxisome-derived ROS affect cellular aging, and are linked to neuroinflammation and to the development of neurodegenerative disorders such as X-ALD, Alzheimer’s disease and multiple sclerosis. The role of peroxisomes in neurodegeration is also supported by the observation that antioxidants help ameliorate symptoms in neurodegenerative disorders such as X-ALD.

All these data imply that peroxisome metabolic activities have central roles in regulating the growing list of metabolites that modulate pro- and anti-inflammatory signaling governing the immune response, thus linking peroxisome activities to the development of immune disorders, chronic inflammatory disease, and neurodegenerative disorders. In addition, the mutations as well as the alteration of the expression level of peroxisomal proteins have gained clinical importance in cancer research [39,40,41,48,67,165]. Future studies to define the extent to which peroxisomes regulate the different innate and adaptive immune cells would be an important step towards understanding how the immune system works, and how peroxisomal immunometabolic activities contribute to the development of immune disorders, metabolic diseases and inflammation. Therefore, studying peroxisomes in immunity and inflammation is highly relevant to human health and disease development. The study of peroxisomes in immunity opens new avenues of investigation regarding the immune modulator role of peroxisomes in inflammatory diseases and offer new opportunities to find novel therapeutic targets to treat infections, chronic inflammatory diseases and neuropathies. Future work can continue to answer more questions to define the functions of peroxisomes in immunity. The exact links between peroxisomal fission and immunity, between peroxisomal activities and immune cell development, the complete identification of peroxisomal metabolites that feed cell and intracellular signaling, the characterization of all antiviral strategies involving peroxisomes, and the detailed understanding of the role of interactions between peroxisomes and mitochondria in immunological signaling are questions that really deserve further study and resolution.

The authors aim with this review to inspire research into many questions that remain to be explored in this new and exciting field of immunity, immunometabolism, inflammation and organelle biology.

## Figures and Tables

**Table 1:** A list of bona fide peroxisomal proteins (**A**) and proteins with an ambiguous peroxisomal localization (**B**) that are expressed in the immune cells and tissues and suggested to participate in the regulation of the immune response.

**A-Proteins with a *bona fide* (strict or partial) peroxisomal localization**					
**Protein**	**Function and Diseases**	**Immune Process**	**Cell or Tissue Type**	Organism *	**Ref**
**ABCD1**	Fatty acid oxidation ABC transporter X-ALD (MIM 300100)	Phagocytosis? Microglial homeostasis? Inflammation	Microglia (BV-2)	m	[56]
			CD14+ monocytes	h	[55]
			Macrophages	h	[55]
			PBMCs	h	[51]
**ABCD2**	Fatty acid oxidation ABC transporter	Phagocytosis? Microglial homeostasis? Inflammation? ABCD1 compensation	Microglia (BV-2)	m	[56]
			Macrophages (peritoneal)	m	[54]
			PBMCs	h	[51]
**ABCD3**	Fatty acid oxidation ABC transporter CBAS5 (MIM 616278)	ABCD1 compensation	PBMCs	h	[51]
**ACOT8**	Fatty acid oxidation Lipid metabolism	Antiviral response Peroxisome proliferation	T lymphoma (Jurkat E6.1)	h	[59]
**ACOX1**	Fatty acid oxidation ACOX1 Def. (MIM 264470)	Phagocytosis? Microglial homeostasis? Inflammation	Microglia (BV-2)	m	[57]
**ACSL6**	Lipid metabolism	Cell proliferation, Leukemogenesis	Bone marrow	h	[35]
			Myeloid leukemia cells	h	[34]
**CAT**	Antioxidant system Acatalasemia (MIM 614097)	Cell proliferation Peroxisome proliferation	Lymph nodes	h	[60]
			T lymphoma (Jurkat E6.1)	h	[59]
			Myeloid leukemia cells U937	h	[44]
			T hybridoma (2B4), B lymphoma A20, mast cell P815	m	[59]
			Microglia (BV-2)	m	[57]
			Macrophages	d	[31]
**DNM1L**	Peroxisome biogenesis Encephalopathy (MIM 614388)	Mitochondrial and peroxisomal fission	Microglia progenitor cells	h	[61,62]
**GNPAT**	Ether lipid synthesis RCDP2 (MIM 222765)	Cell proliferation Maturation of iNKT cells	Thymocytes	m	[32]
**MAVS**	RIG-I-like receptor (RLR) adaptor	Antiviral response	Macrophages	m	[63]
**MFP2**	Fatty acid oxidation MFP2 Def. (MIM 261515)	Inflammation	Microglia (brain)	m	[58]
			Macrophages (Raw)	m	[30]
**NOS2**	Antioxidant system	Brucellosis pathogenesis Inflammation	Dendritic cells and monocytes	m	[64]
			CRL 2471 spleen macrophages	m	[65]
**PEX5**	Peroxisome biogenesis PBD2A (MIM214110), PBDBB (MIM 202370), RCDP5 (MIM 616716)	Inflammation NF-κB regulation	Macrophages	m	[31]
				d	[30,31]
**PEX7**	Peroxisome biogenesis RCDP1 (MIM 215100)	Inflammation NF-κB regulation	Macrophages	m	[31]
				d	[31]
**PEX14**	Peroxisome biogenesis PBD13A (MIM 614887)	Inflammation NF-κB regulation	Macrophages (Raw, primary alveolar, primary peritoneal)	m	[30]
**B-Proteins with an ambiguous peroxisomal localization**					
**Protein**	**Function and Diseases**	**Immune Process**	**Cell or Tissue Type**	Organism *	**Ref**
**FAMIN LACC1**	Fatty acid oxidation	Inflammation	Neutrophils, monocytes/macrophages, dendritic cells	h	[66,67]
			Macrophages (differentiated THP-1)	h	[66,67]
			Lymph nodes, spleen	h	[66,67]
			Macrophages, dendritic cells, neutrophils	m	[68]
**IDH1**	Regeneration of NADPH Lipid metabolism	Hematopoietic differentiation Acute myelogenous leukemia	CD34^+^ bone marrow cells	h	[39]
			Bone marrow mononuclear cells	h	[40]
			Macrophages (THP-1, Kasumi-1, KG-1, OCI-AML3)	h	[41]
**SOD1**	Antioxidant system	Neuroinflammation Cell proliferation	Brain	r	[69]

* h: human, m: mouse, r: rat, d: *Drosophila*.

## Abbreviations

AASA	Aminoadipic semialdehyde
ABC	ATP-binding Cassette
ACOT-8	Acyl-coenzyme A thioesterase 8
ACOX1	Acyl-coenzyme A oxidase 1
AD	Alzheimer’s disease
AMN	Adrenomyeloneuropathy
AMPK	Adenosine monophosphate-activated protein kinase
AMPs	Antimicrobial peptides
cALD	Cerebral adrenoleukodystrophy
CD	Cluster of differentiation
CEL	Carboxyethyl-lyine
CerS	Ceramide synthase
COX	Cyclooxygenase
CNS	Central nervous system
DHA	Docosahexaenoic acid, C22:6 n-3
DAG	Diacyl glycerol
DPA	Docosapentaenoic acid, C22:5 n-3
dsRNA	Double-stranded RNA
EPA	Eicosapentaenoic acid, C20:5 n-3
Fis1	Fission 1 protein
GPX	Glutathion peroxidase
GSA	Glutamic semialdehyde
HCMV	Human cytomegalovirus
HepB/C	Hepatitis B or C virus
HIV	Human immunodeficiency virus
Hnf4	Hepatonuclear factor 4
IFN	Interferon
I-κB	Inhibitor of NF-κB
IKK	I-κB kinase
IL	Interleukin
iNKT	invariant natural killer T cells
IPF	Idiopathic pulmonary fibrosis
LCFAs	Long-chain fatty acids
LPS	Lipopolysaccharide
MDAL	Malonedialdehyde-lysine
MAPK	Mitogen-activated protein kinase
MAVS	Mitochondrial antiviral signaling adaptor
MCFAs	Medium-chain fatty acids
MFP-2	Multifunctional protein-2
MHC	Major histocompatibility complex
MS	Multiple sclerosis
MUFAs	Monounsaturated fatty acids
Nef	Negative regulatory factor
NF-κB	Nuclear Factor kappa-light-chain- -enhancer of activated B cells
NK	Natural killer
NO	Nitric oxide
PBD	Peroxisome biogenesis disorder
PBMC	Peripheral blood mononuclear cells
PEX	Peroxin
PKC	Protein kinase C
PRRs	Pattern recognition receptors
PTS	Peroxisome targeting signal
PUFAs	Polyunsaturated fatty acids
RIG-1	Retinoic acid inducible gene 1
RLRs	RIG-I-like receptors
RNS	Reactive nitrogen species
ROS	Reactive oxygen species
ssRNA	Single stranded RNA
TGF-β	Transforming growth factor beta
TLR	Toll-like receptor
TNF-α	Tumor necrosis factor
TNFR	Tumor necrosis factor receptor
TREM2	Triggering receptor expressed on myeloid cells 2
VLCFAs	Very long-chain fatty acids
vMIA	Viral mitochondria-localized inhibitor of apoptosis
Vps1	Vacuolar protein sorting-associated protein
X-ALD	X-linked Adrenoleukodystrophy
